# Senescent Polarization of Macrophages and Inflammatory Biomarkers in Cardiovascular Disease

**DOI:** 10.3390/cells14171374

**Published:** 2025-09-03

**Authors:** Alojz Ihan

**Affiliations:** Institute of Microbiology and Immunology, Faculty of Medicine, University of Ljubljana, 1000 Ljubljana, Slovenia; alojz.ihan@mf.uni-lj.si

**Keywords:** cardiovascular diseases, aging, macrophages, biomarkers, cell polarization

## Abstract

Cardiovascular diseases (CVDs) are a group of disorders in which inflammatory processes play a crucial role. Age-related chronic systemic inflammation is characterized by elevated levels of inflammatory mediators in the bloodstream. It can occur even in the absence of overt infection, contributing to endothelial dysfunction, vascular stiffness, and atherosclerosis. The regulation of vascular tissue homeostasis and inflammation is primarily mediated by tissue-resident macrophages (TRMs) and monocyte-derived macrophages. The proportion of monocyte-derived macrophages increases with age, contributing to vascular damage and accelerating CVD progression. In aging tissue, monocyte-derived macrophages exposed to various microenvironmental stimuli are predominantly polarized into the pro-inflammatory M1 phenotype. This polarization, in turn, triggers the release of pro-inflammatory cytokines (IL-1β, IL-6, and IL-18) and promotes the generation of oxidative stress molecules. In this review, we examine the role of macrophages in cardiovascular aging, their secretory phenotypes, and the impact of chronic low-grade inflammation on vascular integrity. We also propose reliable biomarkers of chronic cardiovascular inflammation that may aid in risk prediction, patient stratification, and the development of senotherapeutic interventions for cardiovascular disease.

## 1. Introduction

Cardiovascular diseases (CVDs) are a leading cause of morbidity and mortality worldwide, claiming an estimated 17.9 million lives in 2019. While the World Health Organization (WHO) has reported a 27% global decline in age-standardized mortality since 1990, the absolute number of deaths has increased due to population growth and aging. CVDs account for approximately 32% of all global deaths, with over 85% of these attributable to heart attacks and strokes. Notably, about 38% of such deaths occur prematurely (before the age of 70). Age-related changes significantly influence CVD, with older individuals experiencing higher mortality and prevalence rates, although declines in mortality are observed across all age groups [1].

By 2035, CVD is projected to become the most socially costly chronic disease. Although prevention and treatment strategies have improved, many challenges remain unresolved. Therefore, new targets addressing the pathophysiological mechanisms of CVD should be identified [2].

Aging is associated with profound structural and functional changes in the cardiovascular (CV) system. These changes affect both cardiac and vascular tissues and include alterations in cardiomyocytes, fibroblasts, endothelial cells, smooth muscle cells, and extracellular matrix (ECM) components [3].

Cardiomyocytes are terminally differentiated cells responsible for contractile function. Age-related changes include hypertrophy, mitochondrial dysfunction, impaired calcium handling, and accumulation of DNA damage and telomere shortening, which induce senescence or apoptosis and reduce viable cell numbers [4].

Senescence of cardiac fibroblasts leads to dysregulated ECM production and a pro-fibrotic secretory phenotype, characterized by increased collagen I/III deposition and reduced matrix metalloproteinase (MMP) activity. These changes cause increased myocardial stiffness, reduced compliance, and diastolic dysfunction.

Senescent vascular endothelial cells (ECs) promote pro-inflammatory cytokine secretion, adhesion molecule expression, and leukocyte infiltration [5]. Senescent vascular smooth muscle cells (VSMCs) shift from a contractile to a synthetic phenotype, promoting ECM production and calcification and leading to vascular wall remodeling and instability. Senescent extracellular matrix, characterized by fragmented elastin and accumulated collagen, contributes to arterial stiffening [6].

Senescent immune and inflammatory cells progressively infiltrate vascular tissue, causing chronic low-grade inflammation (“inflammageing”) that affects all cardiovascular cell types through increased production of pro-inflammatory cytokines (e.g., IL-6, TNF-α) [7].

The combination of senescent changes—cardiomyocyte hypertrophy, endothelial and VSMC senescence, fibroblast dysfunction, and ECM remodeling—synergistically impairs cardiac and vascular function, increasing the risk of hypertension, atherosclerosis, heart failure, and other CVDs [8,9].

Chronic vascular inflammation, largely driven by age-related changes in vascular immune and inflammatory responses, is a major contributor to CVD. Macrophages play a central role in regulating tissue homeostasis, immune responses, and inflammation, and are a major component of vessel wall infiltrates [10].

Macrophages are major contributors to the pathogenesis of atherosclerosis and inflammatory vasculitides. The mechanisms by which the vascular wall microenvironment influences macrophage differentiation and activation, leading to vascular inflammation, remain poorly understood. This inflammation encompasses a spectrum of diseases, ranging from the slowly progressive wall damage typical of atherosclerosis to vasculitides that can cause life-threatening complications within days to weeks. Inflammatory, tissue-destructive macrophages contribute to the entire spectrum of vessel wall inflammatory diseases [11]. This review aims to present the role of tissue-resident macrophages in cardiovascular aging, their secretory phenotypes, and their role in chronic low-grade inflammation that impact vascular integrity. We also propose reliable biomarkers of chronic cardiovascular inflammation that may aid in risk prediction, patient stratification, and the development of senotherapeutic interventions for cardiovascular disease.

## 2. Macrophages in Cardiovascular Tissue

**Figure 1:**
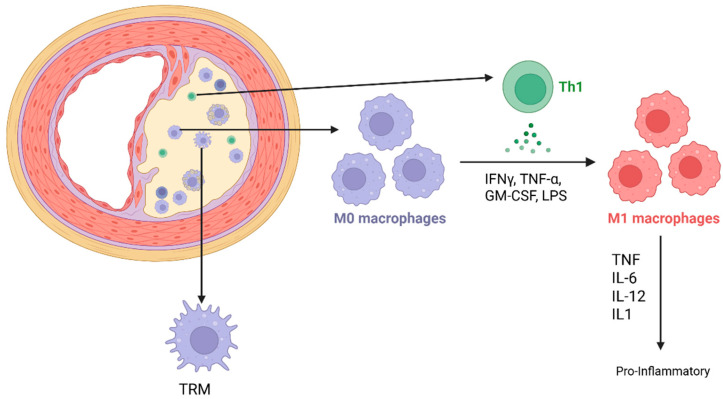
Tissue-resident macrophages (TRMs) and M1 polarized monocyte-derived macrophages, associated with vascular inflammation. TRMs reside in tissues and can rapidly detect environmental changes, responding efficiently to maintain tissue homeostasis. They cooperate with neighboring parenchymal or stromal cells, adopting tissue-specific functions essential for maintaining homeostasis—for example, removing tissue waste products such as dying or dead cells, extracellular matrix components, and subcellular particles like exophers in the heart. M1-polarized monocyte-derived macrophages, linked to vascular inflammation, originate from monocytes recruited during tissue insult to resolve inflammation, clear pathogens, and restore homeostasis. M1 polarization involves activation of intracellular signaling cascades including NF-κB, STAT1, and IRF5, which promote transcription of proinflammatory genes. M1 macrophages express surface markers such as CD80, CD86, and MHC class II, and produce high levels of inducible nitric oxide synthase (iNOS) and reactive oxygen species (ROS). During aging, the proportion of TRMs decreases while the number of monocyte-derived proinflammatory macrophages increases. Figure created with BioRender (biorender.com, accessed on 1 August 2025).

Regulation of cardiovascular tissue homeostasis and inflammation is primarily mediated by tissue-resident macrophages (TRMs) and inflammation-associated monocyte-derived macrophages. TRMs reside in specific tissues, where they rapidly detect environmental changes and respond efficiently to maintain tissue homeostasis. TRMs colonize their target organs early in development from yolk sac progenitors and closely cooperate with neighboring parenchymal or stromal cells, adopting tissue-specific functions essential for organ health and function. TRMs play a critical role in tissue homeostasis by removing waste products, including dying or dead cells, extracellular matrix components, and subcellular particles such as exophers, particularly in the heart [12]. These embryonically derived macrophages can persist for months to years and, in some cases, throughout the organism’s lifespan. They can self-renew locally through low-rate proliferation and do not require continuous replenishment from circulating monocytes, except when damaged by stress events such as acute or chronic inflammation, tissue injury, immune stress, or age-related depletion. In such cases, they are partially replaced by monocyte-derived macrophages. Monocyte-derived macrophages may integrate into the tissue and persist for weeks to months before being replaced by newly recruited monocytes or locally proliferating macrophages. After differentiating into macrophages and occupying tissue niches, monocytes may adopt a resident-like phenotype and, under certain conditions, proliferate locally, thereby extending their lifespan. In the heart, following myocardial infarction, monocyte-derived macrophages can become permanent residents, persisting in tissue for more than six months in experimental mouse models (Figure 1) [12]. 

TRMs are functionally distinct from monocyte-derived inflammatory macrophages, which arise during tissue injury to resolve inflammation, clear pathogens, and restore homeostasis. Inflammatory macrophages typically survive only a few days to weeks. After completing their role, they usually undergo apoptosis, though some may integrate into the tissue and acquire resident-like characteristics. The proportion of inflammatory macrophages increases with age [13]. In mouse studies, TRM self-renewal capacity declines with age, while the number of monocyte-derived proinflammatory macrophages increases, driven by recruitment from cardiac mesenchymal stromal cells [12]. With aging, macrophages develop functional impairments and senescence-associated phenotypes, contributing to chronic inflammation and age-related diseases. Although most senescence studies focus on dividing cells, substantial evidence shows that post-mitotic or terminally differentiated cells, including macrophages, can enter a senescence-like state. In macrophages, this is characterized by a senescence-associated secretory phenotype (SASP), marked by high production of pro-inflammatory cytokines (IL-6, IL-1β, TNF-α), chemokines (CCL2, CXCL8), matrix metalloproteinases (MMPs), and reactive oxygen species (ROS). A pronounced feature of SASP is metabolic reprogramming, including a shift toward glycolysis and mitochondrial dysfunction [12,13].

Aging tissues are influenced by altered macrophage responsiveness and numerous other factors, including changes in the microenvironment, immune activity, tissue injury, hypoxia, and endogenous or exogenous stressors [14]. These changes lead to telomere dysfunction, DNA damage, and activation of cellular senescence, further promoting irreversible growth arrest and a senescent phenotype in tissue cells. In senescent vascular tissue, macrophage motility and environmental sensing are impaired, accompanied by a marked increase in pro-inflammatory activity [15].

Aberrant phenotypes of senescent or damaged vascular cells contribute to tissue fragility and age-related disorders, including autoimmunity, cardiovascular disease, and degenerative conditions. Inflammatory immune diseases are often characterized by altered macrophage phenotypes, which can exacerbate or, in some cases, ameliorate cardiovascular disease. These effects are mediated through activation of NF-κB and NLRP3 inflammasome pathways, triggering the release of pro-inflammatory cytokines (IL-1β, IL-6, IL-18) and generating oxidative stress that promotes vascular injury and atherosclerosis [16,17].

### 2.1. Macrophage Polarization and Plasticity

Macrophages perform diverse functions at various stages of the immune response and inflammation [18]. They express pattern recognition receptors (PRRs) that interact with pathogen-associated molecular patterns (PAMPs), enabling efficient recognition and phagocytosis of pathogens and infected cells, as well as secretion of mediators and inflammatory cytokines to induce an appropriate immune/inflammatory response. Macrophages also function as antigen-presenting cells, effectively bridging innate and adaptive immunity [19,20].

When activated in vitro, monocyte-derived macrophages commit to polarizing into distinct functional subpopulations [21]. Although the M1/M2 polarization paradigm was established in cultured monocyte-derived macrophages, tissue macrophage subsets are also broadly classified as classically activated (M1) or alternatively activated (M2) macrophages. The M1 differentiation program is driven by responses to the proinflammatory cytokine interferon-γ (IFN-γ) and activation of Toll-like receptors (TLRs), such as TLR4 [22]. M2 macrophages mediate diverse regulatory functions, including wound healing, fibrosis, angiogenesis, and immunosuppression [23].

Macrophage phenotypes are shaped by multiple factors, including the microenvironment, immune cell activity, tissue damage, and hypoxia [24]. How infectious or non-infectious danger signals affect the metabolic and functional features of tissue macrophages remains unclear. Tissue aging also contributes to these changes, promoting a chronic low-grade inflammatory state. This promotes significant physiological changes, particularly in cardiovascular tissues [25]. Altogether, age-related inflammatory changes in the cardiovascular system compromise vascular integrity and function, contributing to atherosclerosis, endothelial dysfunction, arterial stiffness, and hypertension [26]. Macrophages exhibit dynamic plasticity and polarization, responding to the microenvironment with functional diversity crucial for vascular health and pathology. Inflammation driven by proinflammatory polarized macrophages can promote cardiovascular disease via excessive activation of the NF-κB and NLRP3 inflammasome pathways, triggering the release of proinflammatory cytokines (IL-1β, IL-6, IL-18) and the generation of oxidative stress molecules. Collectively, these processes promote vascular injury and atherosclerosis [26,27,28,29].

### 2.2. M1 and M2 Macrophage Phenotypes

**Figure 2:**
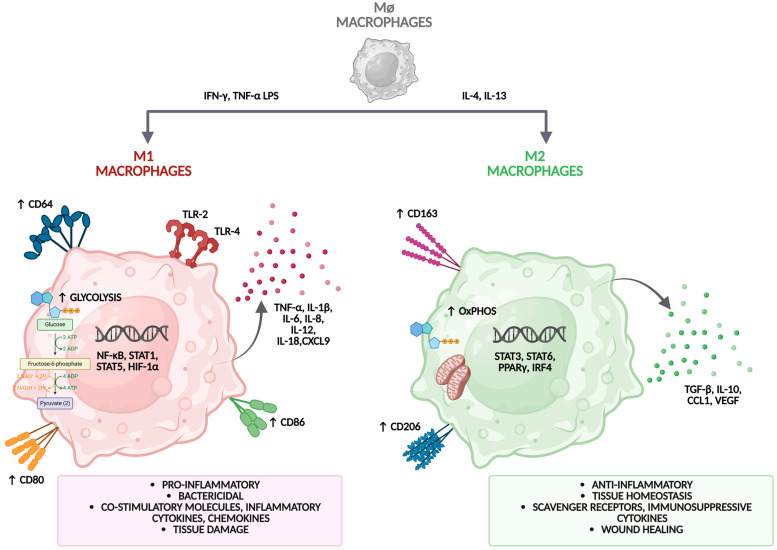
M1 and M2 macrophages. M1 macrophages (classically activated macrophages) arise, when pattern recognition receptors (PRRs), particularly Toll like receptors (TLRs), engage bacterial ligands such as LPS binding to TLR4. This triggers a MyD88 dependent cascade, activating NF κB and MAPK and rapidly inducing transcription of inflammatory genes. M2 macrophages (alternatively activated macrophages) are generated by exposure to pro-inflammatory cytokines such as IL-4, M-CSF, IL-35, IL-13, IL-10, and TGF-β. Unlike classically activated M1 macrophages, M2 macrophages perform a wide range of regulatory functions, including wound healing, fibrosis, angiogenesis, and immunosuppression. Figure created with BioRender (biorender.com, accessed on 1 August 2025).

M1 macrophages, also known as classically activated macrophages, arise upon stimulation with microbial components (e.g., LPS) and IFN-γ. They secrete proinflammatory cytokines such as TNF-α, IL-1β, and IL-6, express inducible nitric oxide synthase (iNOS), and generate reactive oxygen species (ROS). While effective in killing pathogens, they can also exacerbate tissue damage (Figure 2) [22].

M1 macrophages develop primarily in response to microbial stimuli and inflammatory cytokines. For example, exposure to a bacterial TLR ligand such as lipopolysaccharide (LPS) from Gram-negative bacteria polarizes macrophages toward a proinflammatory M1 phenotype. Cytokines such as TNF-α and interferon-γ (IFN-γ), secreted by activated Th1 lymphocytes, have similar effects [23].

M1 polarization involves activation of intracellular signaling cascades, including NF-κB, STAT1, and IRF5, which drive transcription of proinflammatory genes. M1 macrophages express surface markers such as CD80, CD86, and MHC class II, and exhibit high levels of iNOS and ROS production. They are specialized for antimicrobial defense and tumor resistance. Their functions include producing inflammatory cytokines (TNF-α, IL-1β, IL-6, IL-12, IL-23), expressing high levels of MHC II for antigen presentation, and generating nitric oxide (NO) and superoxide to kill microbes. M1 macrophages support Th1 responses and enhance cell-mediated immunity. These responses are critical for controlling acute infections and tumor progression, but sustained M1 activity can contribute to tissue damage and chronic inflammation [21,22].

M1 polarization is induced when pattern recognition receptors (PRRs), particularly Toll-like receptors (TLRs), engage bacterial ligands such as LPS binding to TLR4. This triggers a MyD88-dependent cascade, activating NF-κB and MAPK and rapidly inducing transcription of inflammatory genes. IFN-γ signaling, mediated by the JAK/STAT1 pathway, synergizes with TLR pathways to enhance macrophage activation [27].

Epigenetic mechanisms support M1 polarization, including histone acetylation of proinflammatory gene promoters, reduced DNA methylation of anti-inflammatory genes, and expression of microRNAs such as miR-155 that suppress anti-inflammatory signaling. These modifications establish a transcriptional landscape that sustains the M1 phenotype [28].

M2 macrophages, also known as alternatively activated macrophages, develop in response to cytokines such as IL-4, IL-13, IL-10, and TGF-β. Unlike M1 macrophages, M2 cells mediate diverse regulatory functions, including wound healing, fibrosis, angiogenesis, and immunosuppression [21]. M2 polarization is regulated by transcription factors such as STAT6, PPARγ, and KLF4, which induce expression of genes including arginase-1 (Arg1), chitinase-like protein (Ym1/Chi3l3), found in the inflammatory zone protein 1 (Fizz1/Retnla), CD206 (mannose receptor), and CD163 [22]. M2 macrophages are further classified into subtypes based on induction stimuli and function. M2a cells, induced by IL-4 and IL-13, are linked to tissue repair and fibrosis [18]. M2b cells, induced by immune complexes and TLR/IL-1R agonists, exhibit both anti- and pro-inflammatory activities. M2c cells, induced by IL-10, TGF-β, or glucocorticoids, are strongly immunosuppressive and contribute to matrix remodeling. M2d cells (tumor-associated macrophages, TAMs), stimulated by IL-6 and adenosine, promote angiogenesis and tumor progression. Key signaling pathways driving M2 polarization include STAT6, activated by IL-4/IL-13 receptor engagement, which is essential for M2 gene transcription. PPARγ and PPARδ promote fatty acid metabolism and alternative activation. KLF4 coordinates M2 transcriptional programs while repressing M1 gene expression [30].

Histone modifications (e.g., H3K27 acetylation) and DNA methylation patterns regulate long-term M2 commitment. M2 macrophages depend on oxidative metabolism, fatty acid oxidation, and arginine catabolism via arginase-1, in contrast to the glycolysis favored by M1 cells [31].

In homeostasis, M2 macrophages mediate tissue repair and remodeling. They promote regeneration by clearing apoptotic cells (efferocytosis), producing TGF-β, VEGF, and matrix proteins, and stimulating fibroblasts and epithelial cells for tissue closure. M2 cells also secrete VEGF-A, FGF2, angiopoietins, and MMPs that facilitate vascular sprouting, particularly under hypoxic conditions [32]. M2 macrophages exert predominantly immunosuppressive effects by producing IL-10 and TGF-β, which inhibit proinflammatory T cells and promote regulatory T cell (Treg) expansion [29].

M2 macrophages suppress excessive immune activation in conditions such as inflammatory bowel disease, systemic lupus erythematosus, and rheumatoid arthritis. In obesity, a shift from M1 to M2 phenotype correlates with improved insulin sensitivity and metabolic control. However, prolonged M2 activity can promote fibrotic disorders in the liver, lung, heart, and kidney due to excessive extracellular matrix deposition driven by TGF-β and galectin-3 (Table 1) [31]. 

**Table 1:** M1 vs. M2 macrophage phenotypes.

Characteristic	M1 Macrophages (Classically Activated)	M2 Macrophages (Alternatively Activated)
Activation Stimuli	IFN-γ, LPS (via TLR4), TNF-α [22,23]	IL-4, IL-13, IL-10, TGF-β [21,22]
Signaling Pathways	NF-κB, STAT1, IRF5, JAK/STAT1 [22,23,27]	STAT6, PPARγ, KLF4 [27,30]
Metabolic Profile	Glycolysis, mitochondrial dysfunction [13,26]	Oxidative phosphorylation, fatty acid oxidation [31]
Key Enzymes and Products	iNOS (NO), ROS [21,22]	Arginase-1, VEGF, MMPs [22,32]
Surface Markers	CD80, CD86, MHC class II [21,22]	CD206, CD163 [22]
Cytokines	TNF-α, IL-1β, IL-6, IL-12, IL-23 [22,23]	IL-10, TGF-β [29]
Functions	Antimicrobial defense, Th1 activation, tumor resistance [22,23]	Tissue repair, fibrosis, angiogenesis, immunosuppression [22,29,32]
Role in Disease	Chronic inflammation, atherosclerosis, tissue damage [24,26,33]	Fibrotic remodeling, plaque stabilization, immunoregulation [29,30,32]
Epigenetic Regulation	Histone acetylation, miR-155, DNA demethylation [28]	H3K27 acetylation, DNA methylation, long-term commitment [31]
Subtypes	Not subclassified	M2a (IL-4/IL-13), M2b (ICs + TLR), M2c (IL-10, TGF-β), M2d (IL-6, adenosine) [30,32]

### 2.3. Role of Polarized and Senescent Macrophages in Atherosclerosis

In atherosclerosis, M1 macrophages predominate in unstable plaques, contributing to foam cell formation through internalization of oxidized LDL (oxLDL). They also sustain chronic inflammation, recruit additional monocytes, and promote matrix degradation via matrix metalloproteinases (MMPs), leading to plaque rupture [24]. However, transcriptomic studies suggest that macrophages in vivo span a spectrum of phenotypes, rather than adhering to the strict M1/M2 dichotomy described in in vitro models [12]. In vascular tissues, macrophages may acquire hybrid phenotypes expressing both inflammatory and reparative markers, depending on the stage of atherosclerosis, tissue injury, or repair. In healthy vasculature, M2-like macrophages support particle clearance, smooth muscle cell survival, and extracellular matrix remodeling [13].

Aberrant M1 polarization drives chronic inflammation, endothelial activation, and intimal hyperplasia. In early atherosclerotic lesions, macrophages predominantly polarize toward the M1 phenotype, facilitating LDL oxidation and foam cell formation. In advanced plaques, macrophage death and necrotic core expansion amplify local inflammation. Some macrophages shift toward the M2 phenotype in an attempt to promote tissue repair; however, this transition is frequently inadequate due to a persistently pro-inflammatory microenvironment [32,33].

Senescent macrophages exhibiting the senescence-associated secretory phenotype (SASP) contribute to pathological tissue remodeling by releasing proinflammatory cytokines (IL-6, IL-1β), chemokines (e.g., CCL2), proteases (e.g., MMPs), and reactive oxygen species (ROS).

These mediators promote endothelial activation, leukocyte recruitment, and extracellular matrix degradation. SASP factors from senescent macrophages perpetuate a chronic inflammatory loop, contributing to endothelial dysfunction via reduced nitric oxide (NO) bioavailability and increased expression of adhesion molecules such as VCAM-1 and ICAM-1 [33]. SASP factors also promote plaque progression by enhancing foam cell formation and destabilizing atherosclerotic plaques [34]. Arterial stiffness is exacerbated by increased fibrosis, elastin degradation, and extracellular matrix remodeling. Chronic low-grade inflammation disrupts vascular homeostasis and promotes age-related cardiovascular disease by activating the NF-κB and NLRP3 inflammasome pathways. This results in the release of proinflammatory cytokines (IL-1β, IL-6, IL-18) and the production of oxidative stress molecules. These factors collectively promote vascular injury and the progression of atherosclerosis. This inflammatory state is further exacerbated by dysfunction of immune cells (e.g., macrophages, T cells), and the release of mitochondrial DNA fragments, damage-associated molecular patterns (DAMPs), and reactive oxygen species (ROS) [35].

## 3. Biomarkers for the Assessment of Aging Polarization

In recent years, the understanding of cardiovascular disease has evolved from lipid-centric theories of atherosclerosis to models that recognize chronic low-grade inflammation and immunosenescence as central drivers of disease pathogenesis [35]. Age-related chronic systemic inflammation is marked by elevated levels of inflammatory mediators in the bloodstream, even in the absence of overt infection. This persistent inflammatory state is closely associated with endothelial dysfunction, vascular stiffness, and the development of atherosclerosis.

Identifying reliable biomarkers of chronic cardiovascular inflammation is essential for risk prediction, patient stratification, and the development of targeted anti-inflammatory therapies [36,37].

### 3.1. Chronic Inflammation in Vascular Injury

As individuals age, innate immune cells—such as macrophages, neutrophils, and dendritic cells—become increasingly primed for inflammation. Simultaneously, adaptive immunity exhibits signs of immunosenescence, including reduced diversity in T and B cell repertoires. Aging cells, particularly endothelial cells and macrophages, often develop a senescence-associated secretory phenotype (SASP), characterized by the release of pro-inflammatory cytokines, chemokines, and proteases.

Key signaling pathways involved in the development of SASP include NF-κB activation, NLRP3 inflammasome assembly, JAK/STAT signaling, and increased production of reactive oxygen species (ROS) [38]. These molecular events contribute to endothelial dysfunction, monocyte recruitment, foam cell formation, and plaque instability.

A wide range of biomarkers—including cytokines, chemokines, acute-phase proteins, and immune cell subsets—reflect the complex network of immune activation associated with chronic inflammation and cardiovascular immunosenescence. While traditional markers such as high-sensitivity C-reactive protein (hsCRP) and interleukin-6 (IL-6) remain clinically valuable, emerging technologies such as transcriptomics, proteomics, and extracellular vesicle (EV) profiling hold promise for personalized cardiovascular risk assessment. A deeper understanding of these biomarkers will enhance the prevention, diagnosis, and treatment of age-related vascular diseases [39,40].

### 3.2. Acute-Phase and Soluble Inflammatory Biomarkers

Acute-phase proteins (APPs) and soluble inflammatory markers serve as systemic indicators of immune activation. These proteins are primarily synthesized by the liver in response to proinflammatory cytokines, particularly interleukin-6 (IL-6), IL-1β, and tumor necrosis factor alpha (TNF-α). In the context of aging and cardiovascular disease, where chronic low-grade inflammation contributes to endothelial dysfunction, vascular remodeling, and atherosclerosis, these molecules are valuable biomarkers of disease progression and risk [41,42].

C-reactive protein (CRP) is a prototypical acute-phase reactant produced by hepatocytes under IL-6 stimulation. It binds phosphocholine on dead cells and pathogens, activating the classical complement pathway and facilitating phagocytosis. Clinically, high-sensitivity CRP (hsCRP) is a widely accepted biomarker for cardiovascular risk. Beyond its diagnostic utility, CRP contributes directly to endothelial activation, promotes leukocyte recruitment, and is associated with plaque instability. Elevated hsCRP levels are frequently observed in older adults, even in the absence of comorbidities, indicating an underlying inflammatory state [43].

Serum amyloid A (SAA), another acute-phase protein, is induced by IL-1 and IL-6 and synthesized in the liver. In vascular disease, SAA accumulates in atherosclerotic plaques, promotes foam cell formation, increases endothelial permeability, and enhances matrix metalloproteinase (MMP) release, contributing to plaque destabilization. SAA also acts as a chemoattractant for neutrophils and monocytes. Its chronic elevation in aging is linked to metabolic dysregulation and increased frailty [44].

Fibrinogen, a multifunctional protein involved in coagulation and inflammation, is also synthesized in the liver. Its levels rise in both acute and chronic inflammation. Elevated fibrinogen increases blood viscosity, promotes platelet aggregation, and contributes to thrombus formation on unstable plaques. High fibrinogen concentrations are associated with a greater risk of myocardial infarction, stroke, and peripheral arterial disease. Additionally, fibrinogen interacts with endothelial integrins, promoting cell adhesion and migration. Soluble adhesion molecules in the blood further reflect endothelial dysfunction and inflammatory activity [45].

Adhesion molecules. During vascular inflammation, endothelial cells upregulate adhesion molecules, which are also released into circulation in soluble forms. The most commonly studied include soluble vascular cell adhesion molecule-1 (sVCAM-1), intercellular adhesion molecule-1 (sICAM-1), and E-selectin (sE-selectin). These biomarkers facilitate leukocyte adhesion and transmigration and are elevated in elderly patients with atherosclerosis and heart failure [43].

Pentraxin 3 (PTX3), a long pentraxin structurally related to CRP, is produced locally by macrophages, dendritic cells, and endothelial cells in response to Toll-like receptor (TLR) activation. Unlike CRP, PTX3 reflects localized tissue inflammation. Elevated PTX3 levels are associated with acute coronary syndromes, vascular calcification, and endothelial injury in older individuals [46,47].

Osteopontin (OPN) is a glycoprotein involved in tissue remodeling and immune regulation. Its expression is increased in atherosclerosis, cardiac fibrosis, and vascular calcification. OPN promotes monocyte adhesion, chemotaxis, and MMP expression, and is elevated in older adults, correlating with vascular stiffness and plaque calcification [41].

Soluble CD163 (sCD163) and CD14 (sCD14) are macrophage-derived markers indicative of monocyte/macrophage activation. sCD163, released during hemoglobin clearance, is associated with M2-like macrophage polarization, while sCD14 (also known as presepsin) reflects innate immune activation. Both are elevated in older individuals with cardiovascular comorbidities and are predictive of outcomes in chronic inflammatory conditions and sepsis [46].

Lipoprotein-associated phospholipase A2 (Lp-PLA2) is an enzyme secreted by inflammatory macrophages and bound to LDL particles. It hydrolyzes oxidized phospholipids, generating lysophosphatidylcholine and oxidized fatty acids, both of which are proinflammatory. Clinically, elevated Lp-PLA2 levels are predictive of plaque rupture and ischemic events [44].

Galectin-3 is a β-galactoside-binding lectin secreted by activated macrophages and involved in fibrosis and inflammation. Elevated galectin-3 levels are associated with cardiac fibrosis, heart failure severity, and all-cause mortality in elderly patients. Galectin-3 is also under investigation as both a biomarker and therapeutic target in antifibrotic strategies (Table 2) [46,47].

**Table 2:** Soluble biomarkers of inflammaging and cardiovascular aging.

Biomarker	Function/Role in Inflammaging and CVD	References
C-reactive Protein (CRP/hsCRP)	Acute phase reactant; indicates systemic inflammation and predicts CVD risk	[24,43,46,47]
Serum Amyloid A (SAA)	Promotes foam cell formation, increases endothelial permeability, predicts plaque instability	[44,46,47]
Fibrinogen	Prothrombotic; increases blood viscosity and inflammation, associated with MI and stroke	[43,45,46,47]
sVCAM-1, sICAM-1, sE-selectin	Markers of endothelial activation and leukocyte adhesion	[43,46,47]
Pentraxin 3 (PTX3)	Reflects local vascular inflammation; elevated in acute coronary syndromes	[46,47]
Osteopontin (OPN)	Involved in fibrosis and vascular calcification; predicts arterial stiffness	[41,43,46,47]
Soluble CD163 and CD14	Indicate monocyte/macrophage activation; associated with M2-like phenotype	[46,47]
Lipoprotein-associated Phospholipase A2 (Lp-PLA2)	Promotes plaque rupture and ischemic events	[44,45,46,47]
Galectin-3	Secreted by macrophages; promotes fibrosis and heart failure	[45,46,47]
IL-6	Pro-inflammatory cytokine; induces hepatic acute phase proteins, elevated with age	[45,46,47]
TNF-α	Promotes endothelial activation and chronic vascular inflammation	[45,46,47]
IL-1β	Key cytokine from NLRP3 inflammasome; drives leukocyte recruitment	[45,46,47]
IL-18	Induces IFN-γ, contributes to plaque instability	[45,46,47]
MCP-1/CCL2	Chemoattractant for monocytes; promotes early atherogenesis	[45,46,47]
CXCL8/IL-8	Neutrophil chemoattractant; linked to vascular remodeling	[45,46,47]
IFN-γ	Promotes M1 polarization and plaque vulnerability	[45,46,47,48]
CD14++CD16+ Monocytes	Proinflammatory intermediate monocytes enriched in atherosclerotic plaques	[49,50,51,52,53]
NK Cells (CD56dimCD16+)	Reduced cytotoxicity; increased proinflammatory cytokine production	[49,51,52,53]
Senescent T cells (CD28−CD57+)	Produce TNF-α and IFN-γ; promote inflammaging	[49,51,53]
Treg/Th17 Ratio	Imbalance leads to immune dysregulation and plaque rupture	[54,55,56,57,58]

### 3.3. Cytokine and Chemokine Biomarkers

Cytokines and chemokines are small, secreted proteins that regulate immune responses, facilitate intercellular communication, and orchestrate inflammation. In cardiovascular aging, a persistent proinflammatory state arises from dysregulated cytokine and chemokine production by immune and stromal cells. This inflammatory milieu contributes to endothelial dysfunction, monocyte recruitment, vascular remodeling, and the progression of atherosclerotic plaques.

Interleukin-6 (IL-6) is a pleiotropic cytokine involved in both innate and adaptive immune responses. It is produced by macrophages, T cells, adipocytes, and vascular cells in response to stress and infection. IL-6 activates the JAK/STAT3 signaling pathway and induces hepatic synthesis of acute-phase proteins such as CRP and fibrinogen. IL-6 levels increase with age, reflecting chronic immune activation and metabolic dysregulation. Elevated IL-6 is an independent predictor of mortality in elderly individuals and is associated with increased risk of myocardial infarction, stroke, and heart failure [47].

Tumor necrosis factor alpha (TNF-α) is a proinflammatory cytokine predominantly secreted by macrophages and T lymphocytes. It promotes endothelial activation, upregulates adhesion molecule expression, and induces apoptosis. TNF-α is chronically elevated in older adults and has been implicated in sarcopenia, insulin resistance, and cardiovascular inflammation. High circulating levels are found in patients with atherosclerosis, heart failure, and metabolic syndrome [47].

Interleukin-1β (IL-1β) is a central inflammatory cytokine activated by the NLRP3 inflammasome in response to danger-associated molecular patterns (DAMPs) and cholesterol crystals. IL-1β enhances leukocyte recruitment, increases endothelial permeability, and destabilizes atherosclerotic plaques. It is overexpressed in senescent cells and serves as a hallmark of age-related inflammation. Clinical trials targeting IL-1β have demonstrated significant reductions in cardiovascular events, underscoring its role in disease progression [48].

Interleukin-18 (IL-18), also activated by the NLRP3 inflammasome, stimulates natural killer (NK) and T cells, and acts synergistically with IL-12 to induce interferon-gamma (IFN-γ) production. Elevated IL-18 levels are observed in patients with unstable angina and acute coronary syndrome. In older adults, IL-18 is associated with arterial fragility, vascular stiffness, and increased risk of plaque rupture [47,48].

Monocyte chemoattractant protein-1 (MCP-1/CCL2) is a chemokine essential for recruiting monocytes and macrophages to sites of vascular inflammation. It is produced by endothelial cells, smooth muscle cells, and macrophages, and binds to the CCR2 receptor on monocytes to facilitate their migration and infiltration into the vascular wall. Elevated MCP-1 levels contribute to early lesion formation and the amplification of vascular inflammation [49].

CXCL8/Interleukin-8 (IL-8) is a potent neutrophil chemoattractant and activator produced by endothelial cells and monocytes in response to oxidative stress. IL-8 enhances neutrophil-endothelial interactions, promoting endothelial injury and vascular remodeling. Elevated IL-8 levels are associated with impaired vascular function, chronic inflammation, and poor cardiovascular outcomes [47].

Interferon-gamma (IFN-γ) is a type II interferon secreted by NK cells and Th1 lymphocytes. It promotes M1 macrophage polarization, enhances antigen presentation, and contributes to sustained inflammatory responses. In atherosclerosis, IFN-γ supports macrophage retention within plaques and increases plaque vulnerability. Persistent IFN-γ signaling contributes to chronic immune activation and T cell senescence in aging individuals [47].

### 3.4. Immune Cell Biomarkers in Chronic Inflammation and Immunosenescence of Cardiovascular Aging

Immune cells change significantly during aging and contribute to a chronic inflammatory state known as inflammaging. Dysregulated functions and phenotypic changes in both innate (monocytes, macrophages, dendritic cells, NK cells) and adaptive (T and B lymphocytes) immune cells not only reflect biological aging, but are also responsible for cardiovascular pathologies. The identification of immune cell-based biomarkers of the inflammatory response is crucial for understanding disease progression, monitoring therapeutic interventions and stratifying cardiovascular risk in older populations [50,51,52,53].

A major challenge in identifying suitable markers is the heterogeneity of the senescence phenotype in cardiovascular and immune cells and tissues. Here, multiparameter flow cytometry is a promising and clinically established method. It offers the flexibility to incorporate new surface and intracellular markers and can be used to map immune cell senescence with greater pathophysiological specificity. This in turn allows a more accurate interpretation of systemic inflammatory markers [52,53].

However, despite the great potential of flow cytometry, which is already established in clinical laboratories, it must be acknowledged that its current clinical use is primarily focused on the diagnosis of immunodeficiencies, blood malignancies and autoimmune diseases. Monitoring of age-related changes in immune cells is not part of routine clinical protocols, so there are no recommended and validated markers for defining different age-related immune phenotypes in the elderly [53]. Animal studies show that each type of immune cell has its own characteristic age-related changes and markers. These include different types of cell metabolism and different expression of markers depending on the previous activity of the immune cells in different tissues and in different types of immune responses. It is clear that the described changes cannot be defined solely by the use of established flow cytometric clinical markers for the diagnosis of immunodeficiencies [59].

**Figure 3:**
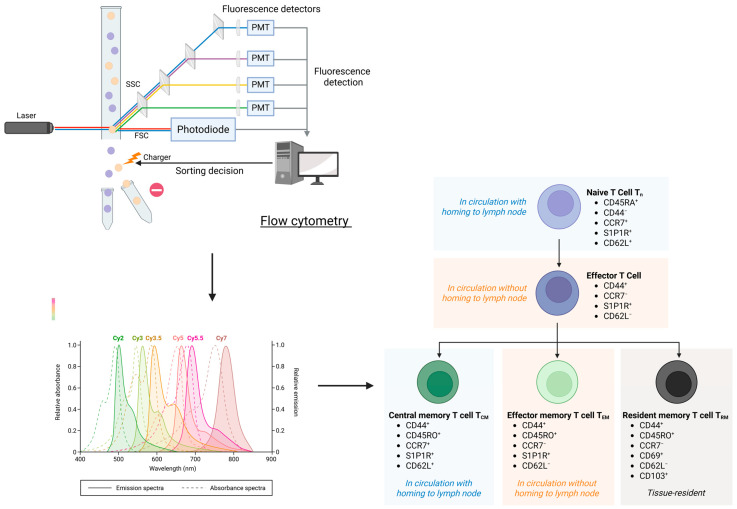
Spectral flow cytometry. Conventional flow cytometry uses multiple detectors, each with a bandpass filter that captures light from a narrow wavelength range. Spectral flow cytometry instead uses an array of detectors (often 32–64 or more) that together capture the entire emission spectrum for each particle or cell across a wide wavelength range. The instrument records the spectral fingerprint of the fluorescence signal, which is the unique emission profile of each dye. Computational algorithms then “unmix” these overlapping spectra to determine the contribution of each fluorochrome to the total detected light. The method enables multiplexing (can use 30–40+ colors in a single sample), can measure the autofluorescence spectrum of cells/tissues and subtract it out, improving signal clarity and is suitable for high-dimensional immune profiling and rare cell detection. Figure created with BioRender (biorender.com, accessed on 1 August 2025).

Spectral flow cytometry improves flow cytometry by resolving the full emission spectra of the cells, allowing up to 50 markers to be analyzed in the same sample. In addition, the spectral approach captures the autofluorescence of a cell population (e.g., macrophages, which are highly autofluorescent) for improved downstream analyses. Given the increasing complexity of heterogeneity of macrophages and other immune cells, it is critical to obtain high-dimensional data at the single cell level to resolve these populations and better define different age-related immune phenotypes in the elderly [53]. New technologies such as artificial intelligence, machine learning, data mining and big data analytics are promising in the optimization of such composite biomarker sets and assess known “classical” clinical markers in combination with newly defined age-related immunophenotypes in the elderly. These tools can be integrated into current models of biological age assessment and cardiovascular risk prediction and, most importantly, improve their ability to detect subtle age-related immunopathological changes (Figure 3).

#### 3.4.1. Innate Immune Cells Biomarkers

Monocytes and macrophages undergo phenotypic remodeling, including inflammaging, during chronic inflammation. Classical monocytes (CD14++CD16−), which make up the majority of the monocyte population, are phagocytic and less inflammatory. In the aging cardiovascular system, an increased proportion of intermediate (CD14++CD16+) and non-classical (CD14+CD16++) monocytes predicts carotid artery intima-media thickness and vascular stiffness. These cells accumulate in atherosclerotic plaques and express high levels of CCR2, CX3CR1 and TLR4. This remodeling is associated with an increase in CD14(low)CD16+ and CD14(high)CD16+ populations and a decrease in CD14(low)CD16− monocytes [52]. Decreased expression of CD62L and TLR1/4 and increased expression of CD11b and TLR5 on macrophages has also been observed, which may promote neoplastic progression [53]. In addition, macrophages exhibit reduced phagocytic activity and shift from the proinflammatory M1 to the M2 phenotype with increasing age. Neutrophils also show a reduced ability to phagocytose pathogens and recruit chemokines, and they become more susceptible to apoptosis. These age-related changes in macrophages and neutrophils contribute to chronic low-grade inflammation and immunosuppression. The number of circulating dendritic cells (DC) also decreases significantly with age [59]. In addition, DC functions such as antigen presentation, endocytosis and interferon-gamma (IFN-γ) production are impaired during immunosenescence, including the ability to activate CD8+ and CD4+ T cells [52,53,59].

**Figure 4:**
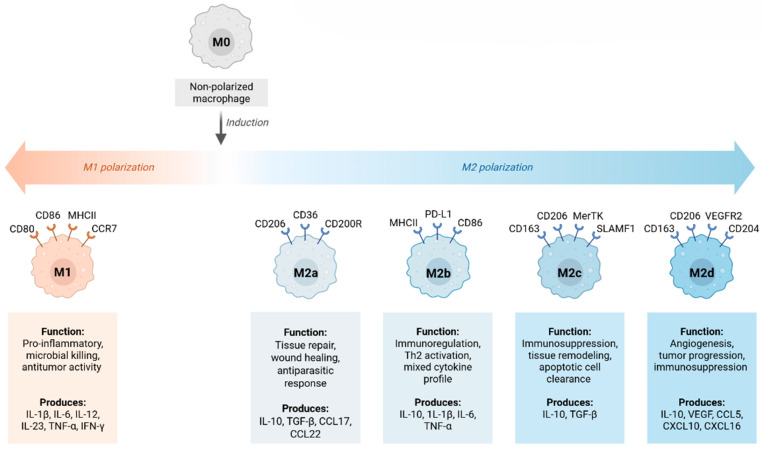
Phenotypic markers of M1 and M2 macrophages. M1 macrophages express surface markers such as CD80, CD86, CCR7, and MHC class II. Their functions include producing inflammatory cytokines (TNF-α, IL-1β, IL-6, IL-12, and IL-23), high expression of MHC II for enhanced antigen presentation, and generating nitric oxide (NO) and superoxide to kill microbes. M2 macrophages are divided into several subtypes based on their induction stimuli and functions. The M2a subtype is induced by IL-4 and IL-13 and is associated with tissue repair and fibrosis. The M2b subtype is induced by immune complexes and TLR/IL-1R agonists and exhibits both anti-inflammatory and pro-inflammatory roles. The M2c subtype is induced by IL-10, TGF-β, or glucocorticoids; these macrophages are strongly immunosuppressive and participate in matrix remodeling. M2d macrophages (tumor-associated macrophages, TAMs) are stimulated by IL-6 and adenosine and promote angiogenesis and tumor progression. The functional roles of M2 macrophages in homeostasis include tissue repair and remodeling. They promote regeneration by clearing apoptotic cells (efferocytosis), producing TGF-β, VEGF, and matrix proteins, and stimulating fibroblasts and epithelial cells to repair the tissue. Figure created with BioRender (biorender.com, accessed on 1 August 2025).

Within macrophages, the M1 subset is proinflammatory and contributes to foam cell formation and plaque instability. M2 macrophages, which have anti-inflammatory effects and promote tissue repair, can nevertheless support fibrosis and angiogenesis under chronic inflammatory conditions. Dendritic cells (DCs), important antigen-presenting cells, mediate communication between the innate and adaptive immune systems through cytokine secretion and T-cell activation [60,61,62,63,64,65]. Although the number and subtypes of DCs do not change significantly with age, their phagocytic capacity is functionally impaired, and their responsiveness to foreign antigens and tolerance to self-antigens decline. In older people, DCs also show a more proinflammatory basal cytokine secretion pattern [63,64]. Myeloid DCs (mDCs) show impaired antigen presentation and T-cell priming, while plasmacytoid DCs (pDCs) show reduced interferon production, which weakens antiviral and antitumor defences. Aging DCs tend to adopt a tolerogenic phenotype and support vascular inflammation. In the clinic, diminished DC function is associated with impaired vaccine response and chronic vascular inflammation (Figure 4).

Natural killer (NK) cells also undergo phenotypic remodeling during chronic inflammation and immunosenescence. Although the number of NK cells remains stable or even increases with age, their cytotoxic function decreases. This change is often characterized by an increase in CD56(dim)CD16+ NK cells with reduced degranulation capacity [66,67,68]. Older NK cells produce higher levels of proinflammatory cytokines and contribute to low-grade inflammation. Elevated levels of granzyme B and perforin are found in dysfunctional NK cells. Similarly, increased circulating levels of NKG2D+ NK cells are associated with endothelial dysfunction. Furthermore, the expression of activating receptors (e.g., NKP30, NKP46 and DNAM-1), which enable NK cells to recognize and lyse tumors, is often decreased, while the expression of inhibitory receptors (e.g., KIR, NKG2C) is often increased during immunosenescence [69,70,71]. Overall, this restructuring of the NK cell profile is characterized by a reduced responsiveness to cytokines, possibly leading to inactivation of dendritic cells and reduced interaction with macrophages.

#### 3.4.2. Markers of Adaptive Immunosenescence

**Figure 5:**
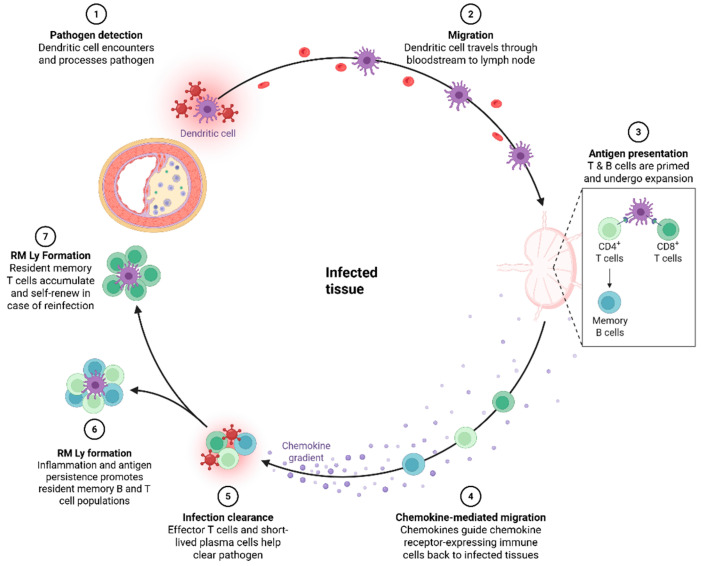
Tissue-resident T and B lymphocytes. Upon detection of pathogens (PAMPs) or tissue damage (DAMPs), dendritic cells actively remove pathogens and dead tissue. They then migrate with the phagocytosed material to regional lymph nodes, where they activate T and B lymphocytes by presenting antigens. This activation leads to the formation of effector T and B lymphocytes, which migrate back to the tissue where dendritic cells initially detected damage. Within the tissue, these lymphocytes are reactivated and mature into memory, resident lymphocytes. After inflammation is resolved, these lymphocytes exit the tissue. When tissues become inflamed again, these cells return and contribute to the acceleration of inflammation. Figure created with BioRender (biorender.com, accessed on 1 August 2025).

A hallmark of adaptive immunosenescence is the accumulation of CD28CD57+ senescent T cells, which secrete high levels of IFN-γ and TNF-α. The decline in naïve CD8+ T cells results from age-related thymic involution [72,73]. This reduction in naïve T cells limits the diversity of T cell antigen receptors and disrupts overall T cell homeostasis. Cytotoxic T cell activity also diminishes with age, reflected in significantly decreased expression of functional effector molecules such as IFN-γ, granzyme B, and perforin [74,75,76]. Furthermore, the numbers of CD4+ and CD8+ regulatory T cells (Tregs) decline with age, impairing the ability to maintain immune tolerance and resolve inflammation. In contrast, proinflammatory Th17 cell numbers increase, and an elevated Th17/Treg ratio has been associated with vascular inflammation and plaque rupture (Figure 5).

Similar to T cells, B cells—which are responsible for antibody production and antitumor responses—also undergo age-related remodeling. The bone marrow niche shifts in favor of myeloid lineage differentiation at the expense of lymphoid cells. Consequently, the circulating levels of naïve CD19+CD27− B cells decline significantly with aging [54,55], a change linked to increased vascular risk. Subsets of mature B cells are redistributed, and their activation becomes impaired with age. The diversity of the B cell receptor (BCR) repertoire is also reduced, likely due to clonal expansion of memory B cells. These age-associated alterations may compromise antibody specificity and increase autoantibody production. In addition, the proportion of CD19+ B cells in peripheral blood decreases with age, accompanied by diminished B cell function. This process is often associated with reduced expression of autoimmune regulators (AIRE) and autoantigen genes in thymic B cells. The regeneration of mature splenic B cells is also frequently impaired in the elderly [54,55,56,57,58].

Senescent macrophages are increasingly recognized as key drivers of chronic inflammation and tissue dysfunction in cardiovascular diseases (CVD), including atherosclerosis, myocardial infarction (MI), and heart failure (HF). These cells exhibit a senescence-associated secretory phenotype (SASP), characterized by the sustained secretion of proinflammatory cytokines, proteases, and chemokines (e.g., IL-6, TNF-α, CCL2), which amplify both local and systemic inflammation. In addition, senescent macrophages display impaired efferocytosis, altered metabolic activity, and resistance to apoptosis—features that contribute to plaque instability and fibrotic remodeling (Table 3).

**Table 3:** Cellular biomarkers of inflammaging and cardiovascular aging.

Biomarker	Function/Role in Inflammaging and CVD	References
CD14++CD16+ Monocytes	Proinflammatory intermediate monocytes enriched in atherosclerotic plaques	[49,51,53]
NK Cells (CD56dimCD16+)	Reduced cytotoxicity; increased proinflammatory cytokine production	[49,51,53,68,70,71]
Senescent T cells (CD28−CD57+)	Produce TNF-α and IFN-γ; promote inflammaging	[49,51,53,68,70,71,72,73]
Treg/Th17 Ratio	Imbalance leads to immune dysregulation and plaque rupture	[54,56,57,58]

## 4. Therapeutic Interventions Targeting Senescent Macrophage Polarization in Cardiovascular Diseases

Senescent macrophages play a key role in the pathogenesis of cardiovascular disease through the persistent secretion of senescence-associated secretory phenotype (SASP) factors such as IL-6, TNF-α, CCL2, matrix metalloproteinases (MMPs) and reactive oxygen species (ROS). Consequently, many therapeutic strategies are being developed to eliminate or modulate senescent cells, including senescent macrophages. Current research in the field of senotherapeutics is focused on two main approaches. The first involves the targeted elimination of senescent cells using senolytics that promote apoptosis in these cells. Examples include quercetin and fisetin (natural flavonoids), navitoclax (an inhibitor of the BCL-2 family) and dasatinib (a tyrosine kinase inhibitor). The second strategy focuses on modulating or inhibiting signaling pathways involved in SASP production, such as the cGAS–STING and NF-κB signaling cascades, or directly targeting proinflammatory cytokines such as IL-1 and IL-6 [77].

Macrophage reprogramming is another promising therapeutic pathway that aims to convert senescent macrophages from a proinflammatory M1-like phenotype to an anti-inflammatory M2-like phenotype to reduce inflammation and support tissue repair [78]. For example, IL-4 and IL-13 therapies have been shown to promote M2 polarization and stabilize atherosclerotic plaques. PPARγ agonists (e.g., pioglitazone) facilitate anti-inflammatory polarization and improve lipid handling. Haemoxygenase-1 (HO-1) inducers promote M2 differentiation and reduce M1-driven inflammation [79]. Another strategy to regulate chromatin accessibility and SASP gene expression is to influence epigenetic changes associated with macrophage senescence and polarization. For example, BET inhibitors can suppress inflammatory transcription programs in senescent macrophages.

Since senescent macrophages exhibit mitochondrial dysfunction and altered energy metabolism, correcting these metabolic abnormalities could reverse senescence characteristics. NAD+ boosters (e.g., nicotinamide riboside) have been shown to restore mitochondrial function, reduce cellular senescence and increase the phagocytic activity of macrophages. Similarly, antioxidants targeting mitochondria can reduce oxidative stress and inhibit inflammasome activation, contributing to further anti-inflammatory effects [80,81].

While many of these therapeutic interventions are still in preclinical development, senolytics and senomorphics (i.e., SASP modulators) are already being tested in clinical trials for age-related cardiovascular disease. Future strategies must carefully balance the elimination and modulation of senescent macrophages to ensure that essential immune functions and tissue repair mechanisms are preserved [81].

## 5. Conclusions

This review highlights the central role of senescent macrophage polarization in age-related chronic vascular inflammation, which is primarily due to age-related changes in the immune and inflammatory responses of vascular tissue. We have also presented a number of clinically relevant biomarkers that can be used to assess cardiovascular aging and inflammation mediated by senescent macrophages and lymphocytes.

As key regulators of immune responses and inflammation, macrophages are central to vascular immune infiltrates and play an important role in tissue homeostasis, waste removal and regeneration. With increasing age, tissue-resident macrophages (TRMs) of embryonic origin—which are normally integrated into tissue homeostasis—undergo apoptosis in response to various stress factors such as acute or chronic inflammation, tissue damage, immune dysregulation or age-related exhaustion. These TRMs are often partially replaced by monocyte-derived macrophages, which can integrate temporarily but are usually short-lived. With prolonged tissue stress, the repeated infiltration of inflammatory macrophages leads to their accumulation and senescence. These senescent macrophages acquire an SASP that contributes to tissue dysfunction and chronic inflammation, ultimately promoting age-related vascular disease.

It is still unclear how systemic infectious or non-infectious danger signals influence the metabolic and functional state of tissue macrophages or how stressed or senescent macrophages promote further tissue damage. Conversely, the effects of metabolic changes in tissues, including senescence, on macrophage function can initiate a vicious cycle of chronic low-level inflammation.

As mentioned above, senescent macrophages develop a SASP characterized by the persistent production of proinflammatory cytokines (e.g., IL-6, IL-1β, TNF-α), chemokines (e.g., CCL2, CXCL8), MMPs and ROS. These changes occur primarily in cardiovascular tissues, which are difficult to access in the clinical setting. However, systemic manifestations of chronic cardiovascular inflammation and altered immune responses can be detected by analyzing soluble biomarkers in body fluids and blood cells.

Although numerous functional studies in animal models have identified highly specific molecular and cellular markers of inflammation, many of them are not clinically validated, often due to the invasive nature of tissue sampling or the complexity of detection methods. In this review, we focus on systemic biomarkers and phenotyping of immune cells in peripheral blood—methods that are commonly used in clinical practice. Most of these biomarkers were originally selected for their strong association with advanced cardiovascular disease in aging populations, but are not always validated for the early detection of age-related cardiovascular changes. As such, they are more applicable in advanced disease stages and less suitable to guide early intervention or treatment monitoring.

To improve risk stratification and monitor response to therapies in aging populations, there is an urgent need for molecular and cellular biomarkers that indicate early-stage vascular damage. This requires the adaptation of study protocols to test known clinical markers for their ability to detect subtle age-related immunopathological changes. As no single molecular or cellular marker can specifically capture the complexity of age-related vascular inflammation, composite biomarker panels need to be developed and standardized.

New technologies—spectral flow cytometry and artificial intelligence—promise to optimize such composite biomarker sets. Given the increasing complexity of heterogeneity of macrophages and other immune cells, it is critical to obtain high-dimensional data at the single cell level to resolve these populations and better define different age-related immune phenotypes in the elderly. These tools can be integrated into current biological aging and cardiovascular risk prediction models and, most importantly, improve their ability to detect subtle age-related immunopathological changes.

## Data Availability

The original contributions presented in this study are included in the article. Further inquiries can be directed to the corresponding author.

## Abbreviations

**Abbreviation**	**Full Term**
APPs	Acute Phase Proteins
CVD	Cardiovascular Disease
TRM	Tissue-Resident Macrophages
ROS	Reactive Oxygen Species
SASP	Senescence-Associated Secretory Phenotype
IFN-γ	Interferon Gamma
TNF-α	Tumor Necrosis Factor Alpha
IL	Interleukin
TLR	Toll-Like Receptor
LPS	Lipopolysaccharide
MHC	Major Histocompatibility Complex
iNOS	Inducible Nitric Oxide Synthase
NO	Nitric Oxide
VEGF	Vascular Endothelial Growth Factor
MMP	Matrix Metalloproteinase
Arg1	Arginase-1
Fizz1	Found in Inflammatory Zone 1
Ym1	Chitinase-Like Protein 3 (Chi3l3)
PPARγ	Peroxisome Proliferator-Activated Receptor Gamma
STAT	Signal Transducer and Activator of Transcription
NF-κB	Nuclear Factor Kappa B
JAK	Janus Kinase
MAPK	Mitogen-Activated Protein Kinase
DC	Dendritic Cell
pDC	Plasmacytoid Dendritic Cell
mDC	Myeloid Dendritic Cell
NK	Natural Killer (cell)
Treg	Regulatory T Cell
Th1	T Helper 1 Cell
Th17	T Helper 17 Cell
BCL-2	B-Cell Lymphoma 2
HO-1	Heme Oxygenase-1
NAD+	Nicotinamide Adenine Dinucleotide
EV	Extracellular Vesicle
CRP/hsCRP	(High-Sensitivity) C-Reactive Protein
SAA	Serum Amyloid A
PTX3	Pentraxin 3
OPN	Osteopontin
sVCAM-1	Soluble Vascular Cell Adhesion Molecule-1
sICAM-1	Soluble Intercellular Adhesion Molecule-1
Lp-PLA2	Lipoprotein-Associated Phospholipase A2
CXCL8	C-X-C Motif Chemokine Ligand 8 (IL-8)
MCP-1/CCL2	Monocyte Chemoattractant Protein-1/C-C Motif Chemokine Ligand 2
DAMP	Damage-Associated Molecular Pattern
PAMP	Pathogen-Associated Molecular Pattern
